# 4-Methylumbelliferone Targets Revealed by Public Data Analysis and Liver Transcriptome Sequencing

**DOI:** 10.3390/ijms24032129

**Published:** 2023-01-21

**Authors:** Alexandra A. Tsitrina, Noreen Halimani, Irina N. Andreichenko, Marat Sabirov, Mikhail Nesterchuk, Nataliya O. Dashenkova, Roman Romanov, Elena V. Bulgakova, Arsen Mikaelyan, Yuri Kotelevtsev

**Affiliations:** 1 Koltzov Institute of Developmental Biology, 26 Vavilov Str., 119334 Moscow, Russia; 2 Ilse Katz Institute for Nanoscale Science & Technology, Ben-Gurion University of the Negev, Beer-Sheva P.O. Box 653, Israel; 3 V. Zelman Center for Neurobiology and Brain Restoration, Skolkovo Institute of Science and Technology, 143025 Moscow, Russia; 4 Department of Molecular Neurosciences, Center for Brain Research, Medical University of Vienna, Spitalgasse 4, A-1090 Vienna, Austria

**Keywords:** 4-methylumbelliferone, nuclear receptors, lipid metabolism, carbohydrate metabolism, RNA-Seq

## Abstract

4-methylumbelliferone (4MU) is a well-known hyaluronic acid synthesis inhibitor and an approved drug for the treatment of cholestasis. In animal models, 4MU decreases inflammation, reduces fibrosis, and lowers body weight, serum cholesterol, and insulin resistance. It also inhibits tumor progression and metastasis. The broad spectrum of effects suggests multiple and yet unknown targets of 4MU. Aiming at 4MU target deconvolution, we have analyzed publicly available data bases, including: 1. Small molecule library Bio Assay screening (PubChemBioAssay); 2. GO pathway databases screening; 3. Protein Atlas Database. We also performed comparative liver transcriptome analysis of mice on normal diet and mice fed with 4MU for two weeks. Potential targets of 4MU public data base analysis fall into two big groups, enzymes and transcription factors (TFs), including 13 members of the nuclear receptor superfamily regulating lipid and carbohydrate metabolism. Transcriptome analysis revealed changes in the expression of genes involved in bile acid metabolism, gluconeogenesis, and immune response. It was found that 4MU feeding decreased the accumulation of the glycogen granules in the liver. Thus, 4MU has multiple targets and can regulate cell metabolism by modulating signaling via nuclear receptors.

## 1. Introduction

In cell culture, 4-methylumbelliferone (4MU) is a coumarin derivative well-known for inhibiting the synthesis of hyaluronic acid (HA). Moreover, 4MU, under the trade name “Hymecromone”, is a prescribed drug for the treatment of cholestasis. Studies in different animal models clearly show that 4MU treatment leads to a wide range of biological effects summarized in reviews [1,2]. Further, 4MU administration inhibits tumor growth and metastasis [3,4,5], fibrosis development [6,7], and inflammation [1,8,9]. A broad spectrum of 4MU biological effects can be explained by inhibiting hyaluronic acid production, but pharmacological targets of 4MU are still under debate. Direct interaction of 4MU and hyaluronan synthase enzyme was not demonstrated. Moreover, 4MU does not inhibit bacterial HAS activity in membrane preparations after the disintegration with ultrasound [10]. Cell-free hyaluronic acid synthesis by membrane preparations from human fibroblasts was not affected by 4MU, too [11]. These experimental facts point towards the possible indirect effect of 4MU on HA synthesis.

It was suggested that 4MU suppresses HA production by depleting the intracellular pool of UDP-glucuronic acid, a precursor of HA [12]. 4MU is a competitive substrate for UGT-glycosyltransferase (UGT), an enzyme which generates precursors for HA synthesis, UDP-glucuronic acid (UDP-GlcA) and UDP-N-acetylglucosamine (UDP-GlcNAc). Recently published data suggested that this interpretation may be insufficient. Nagy et al. demonstrated that in vivo administration of 4MU significantly decreased the level of UDP-GlcA but did not reduce the total concentration of HA in mouse pancreas, muscle, and liver [13]. The fact that 4MU does not inhibit chondroitin sulfate and heparan sulfate synthesis, where both HA precursors are involved, contradicts the UDP-GlcA depletion hypothesis [14,15]. According to published studies and the data from our laboratory, 4MU exerts a dual action on mammalian cells: at low micromolar concentration, it inhibits the HA synthesis, but in concentrations higher than 200 μm, it strongly affects multiple gene expressions, including HAS2 [6,15,16]. Our transcriptomic analysis demonstrated strong effects of 4MU on gene expression in hepatocytes, the cells which do not express HAS enzyme [6]. All these data point to multiple biological effects of 4MU.

As mentioned in many papers, 4MU treatment has a significant influence on the immune response [17,18]. Moreover, 4MU treatment decreases inflammation in mouse models of type 1 and 2 diabetes [19,20,21], multiple sclerosis [22], autoimmune arthritis [23], acute lung injury [18], liver fibrosis [6], and many others. Further, 4MU inhibits antigen presentation by dendritic cells [24] and blocks T cell proliferation activation and Th1 polarization [17,25]. In addition, 4MU primes Th2 polarization and induction of Foxp3+ regulatory T cells [17]. Treatment with 4MU also reduces macrophage accumulation in the fibrotic area [6,7], switches hepatic macrophages to proinflammatory M1 phenotype, and reduces the aggressiveness of hepatocellular carcinoma [26].

Recently published data also indicates that 4MU administration induces a metabolic switch in different cell types, and this effect is not directly linked to HA inhibition. It was shown that 4MU treatment inhibits glycolysis in chondrocytes [8,9] and melanoma cell lines [27]. At present direct biological targets and mechanistic explanations of the biological effects of 4MU are missing. This hampers the development of new, more specific, and more potent drugs than hymecromone.

Here, we present an analysis of publicly available data bases: 1. Summary of 4MU targets identivied by small molecule libraries screening, depositted in PubChemBioAssay; 2. GO pathway analysis of identified 4MU target proteins; 3. Analysis of 4MU target protein expression across various human cell types (Single Cell and HPA datasets from Protein Atlas). Database analysis and our experimental data reveal that 4MU has multiple targets and can regulate cell metabolism through the modulation of nuclear receptor signaling.

## 2. Results

### 2.1. Analysis of 4 MU Targets and Open Access Data Bases

#### 2.1.1. Potential 4MU Biological Targets Revealed by PubChem BioAssay Data

**Figure 1:**
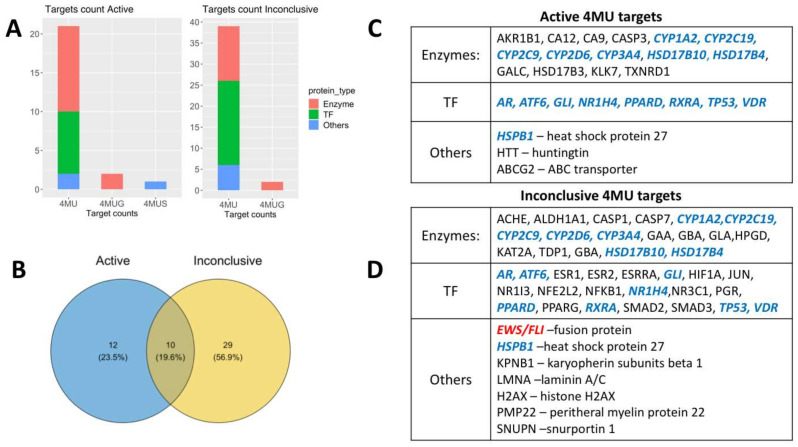
4MU, 4MUG and 4MUS targets retrieved from PubChem BioAssay database. (**A**)—target counts in Active and Inconclusive data sets with respect to target function and 4MU derivative. (**B**)—Venn diagram with Active and Inconclusive sets intersection. (**C**)—List of Active targets, (**D**)—list of Inconclusive targets. Common targets for Active and Inconclusive sets are shown in blue, and EWS/FLI protein, which was excluded from further analysis, is shown in red.

As of 30 May 2022, there were 1,465,985 bioassays deposited on the PubChem BioAssay database (https://pubchemdocs.ncbi.nlm.nih.gov/bioassays; accessed on 30 May 2022) including 111,398,703 compounds, encompassing 103,628 genes and 185,202 proteins [28]. The dataset for analysis was collected manually from the following queries: 4-methylumbelliferone (4MU, 1676 records), 4-methylumbelliferyl-beta-D-glucuronide (4MUG, 71 records) and 4-methylumbelliferyl sulfate (4MUS, 15 records), all henceforth jointly referred to as 4MU targets. Each record describes the effect of 4MU observed in small molecule libraries screening experiments targeting a particular protein or process activity. We excluded entries where 4MU did not have any activity, partially curated data records and experiments dedicated to the antibacterial properties of 4MU. After parsing the initial targets through the exclusion criteria, the 51 records obtained were sorted into either Active or Inconclusive sets based on PubChem BioAssay activity score. The final result after deduplication of 4MU and 4MUG common targets (HSD17B4, HSD17B10 for “Active” and GBA, HPGD for “Inconclusive”) contained 22 “Active” targets and 39 “Inconclusive” targets 10 of which were common in both sets (Figure 1B). Using the Gene Ontology database, we clustered the target proteins by function into three main groups namely: Enzymes (23 records), Transcription factors (TF; 20 records), and Others (nine records), mainly transport and scaffold proteins, as shown in Figure 1C,D and Table 1. 

#### 2.1.2. 4MU Target Proteins Revealed by Analysis of Affected Molecular Pathways

**Figure 2:**
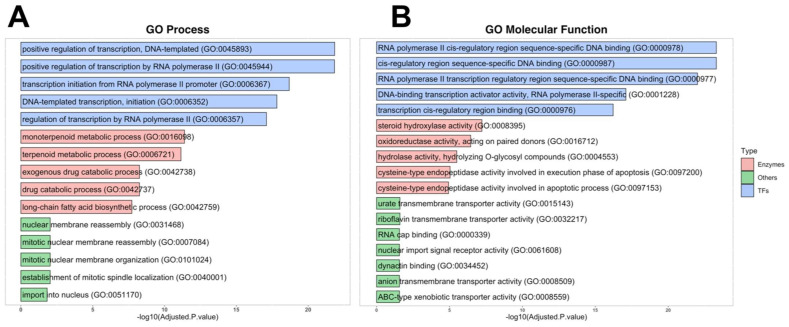
Main GO ontologies and pathways of 4MU targets. (**A**)—GO Process ontologies, (**B**)—GO Molecular Function.

For the investigation of the involvement of 4MU target proteins in biological processes and pathways, we used the GO Database terms. TFs were mainly identified using DNA-templated RNA transcription through cis-regulatory regions. Among identified TFs, 13 belong to the superfamily of nuclear receptors binding lipophilic ligands. The Enzymes group included enzymes primarily involved in steroid hormone metabolism, monoterpenoid metabolic processes, exogenous drug metabolism, long-chain fatty acid biosynthetic processes, and apoptosis. These enzymes possess hydroxylase, oxidoreductase, hydrolase and caspase activities “Others” group included proteins involved in nuclear membrane reassembly, organisation, and nuclear import with transmembrane transporter activity, RNA cap, and dynactin binding (Figure 2A,B).

The type of quantitative High throughput screens performed (qHTS) are highlighted and were accessed in PubChem BioAssay using the AID numbers shown in Table 2.

Treatment with 4MU activates cellular stress response pathways mediated by TP53, HIF1A, NFE2L2, and ATF6 and initiates NFkB-dependent transcription (Table 2). It also activates RXRA, the main dimerization partner for type I nuclear receptors, and mediates the biological effects of retinoic acid. Such pattern of TFs indicates activation of the defence program against different stress stimuli followed by a shift in fatty acid and glucose metabolism. 4MU has a dual effect (activation and inhibition) on 12 TFs (Table 2), mainly nuclear receptors. Furthermore, 4MU elicits different responses depending on the cell line and experimental conditions as is the case with VDR and NR3C1 (glucocorticoid receptor) which are well-known modulators of the immune response.

In addition, 4MU stimulated the activity of enzymes, such as CASP3, GALC, KLK7, GAA, and GBA, which modulate protein processing, cell motility, membrane glycolipid composition and glycogen breakdown. In this Enzymes group, KLK7 peptidase was activated by the lowest concentration of 4MU (7 µM). KLK7 peptidase is involved in skin shedding, cancer and Alzheimer’s disease progression [90,91,92].

It was found that 4MU inhibited 18 out of 24 enzymes (Table 2). These enzymes are involved in lipid and drug metabolism, oxidative stress response, cytokine activation, transcription and DNA repair processes. CA9 was inhibited by the lowest concentration of 4MU (0.56 μM). CA9 is abundantly expressed in many cancer types where it maintains the normal pH level in tumor cells in the hypoxic environment [93].

Transcription activity of GLI and NR1I3 and nuclear imports mediated by SNUPN and KPNB1 from the other groups was also inhibited by 4MU. Interestingly, we found three proteins in this group specifically involved in the development of inherited neurological disorders:

(i) Huntingtin protein (HTT) is involved in axonal transport. The mutation of the Huntingtin gene causes Huntington’s disease. Treatment with 4MU leads to cytoprotection in the cell model of Huntington’s disease (PubChem bioassay AID 1471).
(ii) PMP22 is integrated into the myelin sheath of Schwann cells and is involved in Charcot–Marie–Tooth disease. Treatment with 4MU inhibits the PMP22 expression in Swann cells.
(iii) Nuclear Lamin A (LMNA), a nuclear envelope scaffold protein involved in chromatin organisation, remodelling, and double stranded break DNA repair [94]. Mutations in LMNA cause the development of Hutchinson-Gilford syndrome, characterised by early premature ageing. Moreover, 4MU modulates the expression of Lamin A (PubChem Bioassay 1487).

In vitro, 4MU activated the phosphorylation of H2AX on Ser-139, a sensitive marker of DNA double strand break [95], and stimulated the expression of HSPB1, indicating the activation of the heat shock response pathway. These data correlate with ATF6α activation, which is the main sensor of unfolded protein stress in ER [58]. All these proteins from the “Others” group (HSPB1, LMNA, HTT, PMP22 and H2AX) can be considered indirect targets of 4MU.

#### 2.1.3. 4MU Target Proteins Revealed by Analysis of Gene Expression in Various Human Cell Types

**Figure 3:**
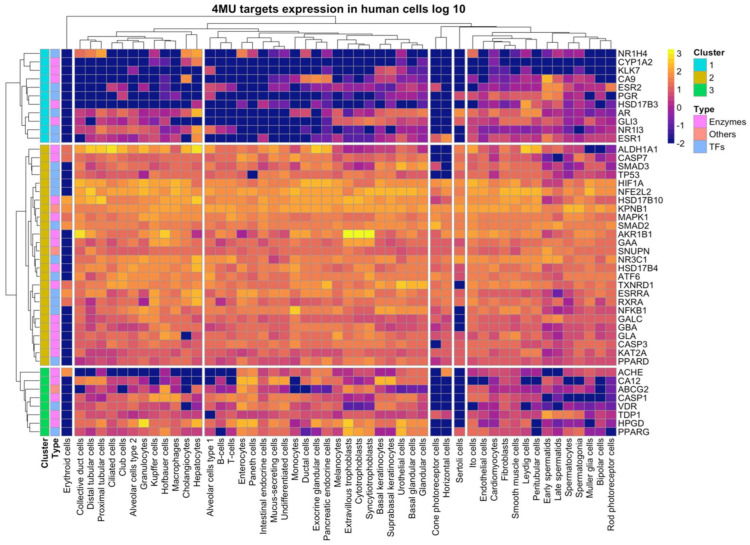
4MU targets expression across human cell types. The expression levels of individual genes were retrieved manually from the Single Cell RNA-Seq dataset of the Human protein atlas database (www.proteinatlas.org). Data presented in log 10 scale, −2 values correspond to the undetectable expression level.

The target gene expression of 4MU was evaluated in 51 human cell types from the Single Cell data of the Protein Atlas Database. The target genes 4MU fell into three clusters. Cluster 1 comprised of 11 genes; NR2H4, CYP1A2, KLK7, CA9, ESR2, PGR, HSD17B3, AR, CLI3, NR1I3, and ESR1. Expression of these genes varied significantly across cell types. Cluster 2 consisted of 26 genes, all highly expressed in almost all cell types. Cluster 3, composed of eight genes, namely ACHE, CA12, ABCG2, CASP1, VDR, TDP1, HPGD, and PPARG, showed a moderately high expression in some cell types. All gene clusters were detected in the cardiomyocytes in contrast to erythroid cells in which gene expression was undetectable (Figure 3). These data indicate that 4MU alters metabolic pathways in a vast majority of cell types.

**Figure 4:**
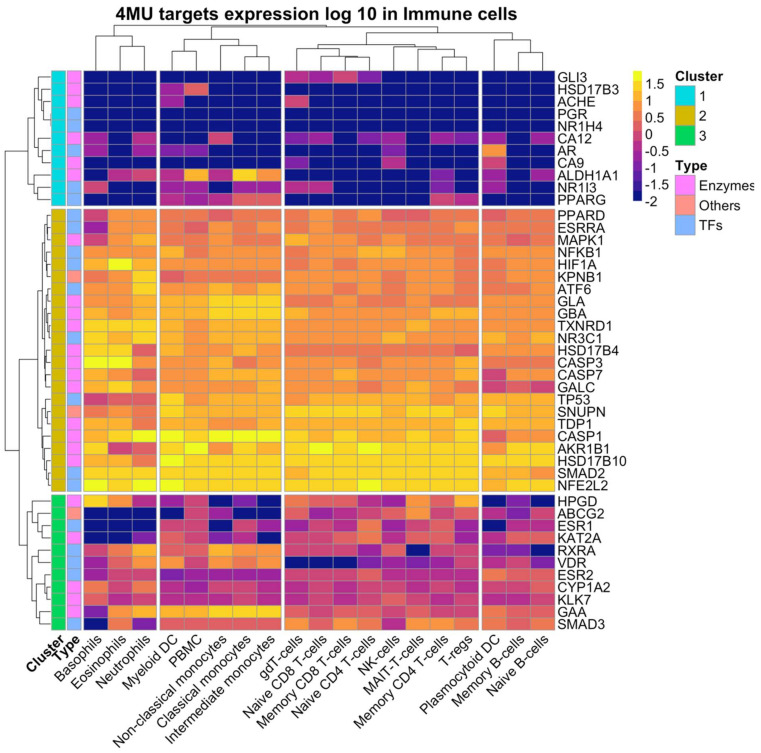
Expression of 4MU targets in the immune cells. Data were retrieved manually from the HPA RNA expression dataset from the Human Protein Atlas database. Data present in log 10 scale, −2 value represent the absence of expression.

We also analysed 4MU target gene expression in immune cells using the data from the HPA dataset deposited in the Protein atlas database. Immune cells express 43 out of 45 genes which form three clusters. PGR and NR1H4 expression was undetectable (Figure 4).

Cluster 1 includes eight genes whose expression is generally low and is specific to peripheral blood mononuclear cell (PBMC), non-classical, classical, and intermediate monocytes. The co-expression of ALDH1A1, NR1I3, and PPARG is characteristic of PMBC, myeloid dendritic cells (DC), macrophages, and memory CD4+ T cells. CA12, CA9, AR, ALDH1A1, and NR1I3 co expression is unique to plasmacytoid DC.

Cluster 2 contained 26 genes with ubiquitous and high expression across all analysed cell types. The group of TFs was comprised of ATF6, ESRRA, HIF1A, SMAD2, NFE2L2, NFKB1, PPARD, TP53, and NR3C1 and the enzymes group consisted of AKR1B1, HSD17B4, HSD17B10, CASP1, CASP3, CASP7, CYP1A2, GALC, GBA, GLA, TXNRD1, and TDP1.

Cluster 3 contained 11 genes expressed in most analysed cell types (Figure 4). This cluster included HPGD, ABCG2, ESR1, KAT2A, RXRA, VDR, ESR2, SMAD3, CYP1A2, GAA, and KLK7. The expression levels of cluster 3 genes were lower than those of cluster 2 genes (Figure 4).

Based on the target annotation above, we postulate that 4MU influences lipid metabolism, cytokine production, antioxidant activity and apoptosis in immune cells. NFE2L2 is a 4MU target, which is highly expressed in lymphocytes. This TF upregulates the transcription of many antioxidant and cytoprotective proteins, including TXNRD1 [96]. NFE2L2 signalling pathway antagonises NFkB-pathway regulating expression of the genes involved in inflammatory, immune and acute-phase responses [97,98]. Furthermore, NFE2L2 inhibits TGF-β signalling and HIF1-mediated immune response [99,100].

Earlier on, we highlighted that the type of response to 4MU elicited is dependant type of cell line and experimental conditions as is the case with VDR and NR3C1. NR3C1 is highly expressed in all immune cells and regulates innate and adaptive immunity. Generally, NR3C1 suppresses proinflammatory activation of macrophages, inhibits the production of cytokines (IL-1, IL-6, IL-8) and chemokines (Ccl2, Ccl3, Ccl4, Cxcl9, and Cxcl11) which promotes the acquisition of inflammation resolving phenotype [70]. Functional activity of NR3C1 is essential for T-cell polyclonal activation and maturation of either Th1- and Th2- cell types [101]. It is also involved in the modulation of B-cell apoptosis [102].

VDR is a potent immune stimulator, upon activation boosts the innate immune cells’ chemotactic and phagocytic capabilities. Moreover, VDR induces the transcription of cathelicidin and defensin *β*2 [78]. It was shown that VDR activation prevents nuclear translocation of p65/p50 subunits of NFkB and degrades IκBα protein, thereby inhibiting the inflammatory response [103]. Its activation suppresses autoimmunity driven by Th1 and Th17 cells [78]. The mild immunosuppressive properties of 4MU can be explained by the synergistic action of both VDR and NR3C1, which occurred in the 30–50 μM range according to PubChem BioAssay data analysis (Table 2).

### 2.2. Experimental Analysis of 4MU Effect on Liver Transcriptome and Glycogene Storage

#### 2.2.1. Effect of 4MU Treatment on the Gene Expression Profile of Normal Mouse Liver

According to the previously published data, the liver is one of the most prominent target organs of 4MU action [13]. Our previously published results demonstrated a profound effect of 4MU on gene expression after two weeks of treatment and its cessation after four weeks in CCL4 induced murine model of hepatic fibrosis [6].

**Figure 5:**
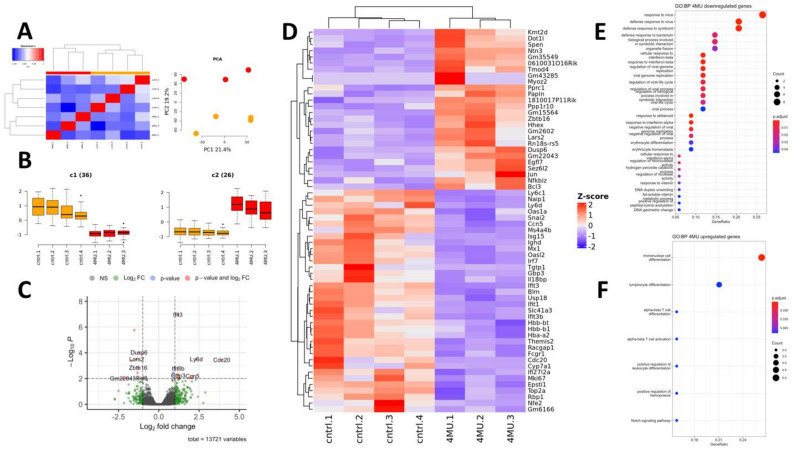
Characterisation of 4MU differentially expressed genes. (**A**)—Correlation between control and 4MU-treated samples. (**B**)—Mean expression of down- and upregulated genes across the samples; (**C**)—Volcano plot of differentially expressed genes; (**D**)—Heatmap of DEGs. (**E**)—Pathway enrichment analysis of down-regulated genes; (**F**)—Pathway enrichment analysis of upregulated genes; Gene ratio—the number of DEG which falls into the specific pathway to the total number of genes in this pathway. Symbol meaning 6B: c1(36) the number of down regulated genes (*n* = 36) in 4MU group (4MU.1, 4MU.2, 4MU.3) in comparison with control group (cntrl.1, cntrl.2, cntrl.3, cntrl.4′); c2(26) the number of up regulated genes (*n* = 26) in 4MU treated group.

In this study, we present a detailed transcriptomic analysis of 4MU-dependent changes in a healthy liver. Bulk RNA-Seq data analysis showed a high degree of similarity between control and 4MU treated liver samples, as reflected by Spearman correlation coefficients (Figure 5A). Principal component analysis (PCA) showed that 40.6% of sample variance originated from the first two components, which clearly separated the experimental groups into two clusters (Figure 5A). Oral 4MU treatment of healthy animals has very mild effects on gene expression. We found 62 genes which were significantly up- (26 genes) and downregulated by 4MU (36 genes), (Figure 5B). The complete list of DEGs with their relative expression levels is presented in Figure 5C,D and Table 3. Affected genes were annotated according to GO Biological Pathways database terms (Figure 5E,F). Most of the downregulated genes (29 out of 36) are involved in different aspects of immune response (Figure 5E). These genes encode the surface markers of different immune cells: *Ly6d* is a marker of B and dendritic cells, *Ly6c1, Themis2*, and *Ms4a4b,* are markers of T and NK cells; whilst *Fcgr1* is characteristic of liver macrophages. Treatment with 4MU significantly decreases the expression level of 14 genes involved in interferon signalling and response (*Tgtp1, Mx1, Epsti1, Ifit1, Ifit3, Ifit3b, Gbp3, Isg15, Il18bp, Usp18, Irf7, Oasl2, Ifi27l2a, Oas1a*). The expression level of *Naip1* was also significantly diminished by 4MU. *Naip* 1, whose role is to recognise bacterial proteins, is abundantly expressed in dendritic cells and macrophages.

In the 4-MU treated mice, we observed a decreased expression of proteins (*Cdc20, Mki67, Ccn5, Racgap1*) involved in cycle progression, and maintenance of DNA integrity and stability (*Top2a, Blm*). Among other downregulated genes, we identified two transcriptional factors (*Nfe2, Snai2*), haemoglobin’s chains genes (*Hba-a2, Hbb-b1, Hbb-bt),* retinol-binding protein 1 (*Rbp1*), Ig delta chain C region (*Ighd*) and mitochondrial Mg^2+^ transporter (*Slc41a3*). Surprisingly, Cyp7a1 one of the critical enzymes of bile acid production was downregulated four-fold [104].

The upregulated genes are involved in the modulation of transcriptional activity (*Bcl3, Spen*), histone acetylation (*Dot1l, Kmt2d*), protein phosphatase activity (*Dusp6, Ppp1r10*), mitochondrial biogenesis and function (*Lars2, Pprc1*), extracellular matrix remodelling (*Egfl7, Ntn3, Papln*), muscle cells differentiation (*Myoz2, Tmod4*), and cancer progression (*Sez6l2*). Moreover, 4MU induced a nearly two-fold increase in the expression levels of transcriptional factors *Hhex, Jun, Nfkbiz,* and *Zbtb16 (*Figure 5C,D; Table 3). Hhex is an essential transcription factor for hepatoblast differentiation and intrahepatic bile duct morphogenesis [105]. Embryonic knockout of Hhex is lethal because it causes abnormal liver development. In adult mice, transcriptional activation of Hhex expression depends on Farnesoid X receptor (NR1H4) activation by bile acid. Overstimulation of this signalling pathway leads to liver hypertrophy [106]. Jun is involved in hepatocyte proliferation and liver regeneration [107]. It has been demonstrated that c-Jun is involved in the bile-acid-induced downregulation of Cyp7a1 expression [108]. Nfkbiz is a transcriptional regulator that augments the inflammatory responses from Toll-like receptors or interleukin signalling. The hepatocyte-specific knockout of this gene significantly accelerates the progression of non-alcoholic fatty liver disease in mice, while its overexpression protects the liver from steatosis. Microarray analysis revealed that the overexpression of Nfkbiz downregulates the expression of genes involved in the triglyceride metabolism pathway [109]. Zbtb16, also known as promyelocytic leukaemia zinc finger protein (PLZF), is a transcription repressor involved in energy metabolism maintenance and pathogenesis of metabolic diseases. In the adult liver, Zbtb16 is an essential regulator of gluconeogenesis. The activation of Zbtb16 expression induces the expression of vital gluconeogenic genes in vitro and in vivo [110]. Zbtb16 expression is activated by the glucocorticoid receptor (NR3C1) in many cell types, including hepatocytes [110], breast cancer cell line [111], endometrial stromal cells, and myometrial smooth muscle cells [112].

**Figure 6:**
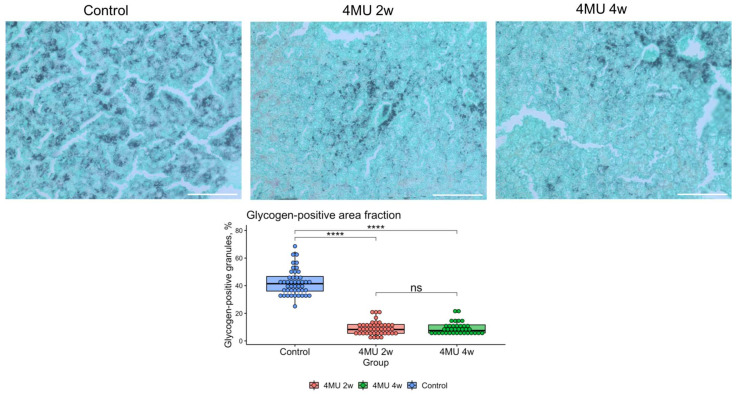
Glycogen granules in mouse hepatocytes after 4MU treatment. Gomori-Grocott methenamine silver staining of glycogen granules in mice hepatocytes after 2 (2w) and 4 weeks(4w) of 4MU treatment. In the figure, hepatocytes are light green and dark grey while glycogen granules are black. Scale bar—100 μm. The plot represents the percentage of glycogen-positive area to total tissue area. Statistical difference was estimated by one-way ANOVA with Tukey post-hoc test (****—*p*.val < 0.0001; ns—not significant).

This finding is in concert with the observed depletion of glycogen granules in mice after treatment with 4MU, as shown in Figure 6.

#### 2.2.2. Effect of 4MU on Glycogen Storage in the Liver

Public data analysis (Pub Chem Bio Assay, Table 2 assay id 2112) shows 4MU activation of acid alpha-glucosidase (GAA), a key enzyme involved in lysosomal glycogen metabolism. The half-maximal efficacy concentration of 4MU for GAA was 25.1 μM in vitro (Table 1 and Table 2). We performed glycogen granules staining with Gomori-Grocott methenamine silver stain in healthy liver at two or four weeks after 4MU treatment *n* = 3 controls, *n* = 4 in 4 MU treated group. As anticipated, GAA activation by 4MU depleted hepatocyte glycogen granules after two weeks (Figure 6). However, the glycogen granules increased after four weeks.

## 3. Discussion

There are two major processes of drug discovery, namely target-based drug discovery (TBDD) and phenotype-based drug discovery (PBD). TBDD is a predominant route taken by pharmaceutical companies developing drugs interacting with well-characterised targets. In contrast to TBDB, PBD evaluates a compound’s ability to modulate a certain trait of a given biological system, the effect of a biologically active compound is based on the analysis of cell culture and whole-body phenotypes. Initially, the biological target and molecular mechanism of action are unknown. Nonetheless, effective drug development demands we identify the drug targets of the biologically active compounds.

In this study we identified 4MU targets by analysing data from publicly available databases coupled with our RNA-Seq transcriptomic profiling of bulk liver. We show that 4MU modulates the functional activity of at least 45 proteins, mainly TFs and enzymes. These proteins are expressed in 51 different human cell types, including immune cells, which explains the wide range of biological effects upon 4MU treatment. According to the data, 4MU modulates the activity of at least 13 nuclear receptors, namely the transcription factors which regulate various metabolic pathways and immune responses. Our analysis of publicly available data indicates that 4MU treatment leads to activation of cell stress response pathways via HIF1A, NFkB, NFE2L2 and ATF6 transcription factors. In addition, 4MU exposure activates several lysosomal enzymes involved in the degradation of glycolipids and glycogen (GAA, GBA, GALC and GLA).

In our previously published work [6], we showed that 4MU inhibits HAS2 expression and hyaluronan deposition in liver parenchyma during the development of liver fibrosis in mice. In the current study, we evaluate the effect of 4MU treatment in normal liver, which expresses very low levels of HAS2 [113]. Our data indicate that 4MU influences energy metabolism in a healthy liver by activating glycogen utilisation. Exposure of mice to 4MU for two weeks led to the depletion of glycogen granules in the liver. These findings are consistent with publicly available data of high content screening experiments targeting GAA, the main glycogen catabolic enzyme. The exhaustion of glycogen indicates an alteration in the liver’s energy and carbohydrate metabolic signature. This finding is in correlation with the conclusion of the recently published extensive research on the effects of 4MU on energy metabolism in mice [20].

Liver transcriptome analysis of mice on normal and 4MU diet confirms this finding: 4MU strongly affected the expression of Zbtb16, one of the key regulators of hepatic gluconeogenesis [110]. Concurrently, 4MU treatment significantly affected the expression of genes involved in bile acid metabolism. Even though 4MU was introduced in some countries as a drug for cholestasis treatment more than 30 years ago, the precise mechanism of its action remains unknown. Our findings reveal an upregulation of Hhex and Jun, the transcription factors regulating the conversion of cholesterol into bile acids and a downregulation of Cyp7a1 which is the rate-limiting enzyme in bile acid synthesis via the classical pathway [104,106,108]. The expression of Cyp7a1 is tightly regulated; it is induced by feeding and suppressed by high levels of bile acid in the serum through activation of NR1H4 (Farnesoid X receptor). Such an autoregulatory loop controls the enterohepatic circulation of bile acids and maintains a constant circulating level of bile acid [114]. It has been shown that bile acid regulates the expression of c-Jun via nuclear receptor SHP [115], and c-Jun is involved in NR1H4-dependent downregulation of Cyp7a1 [108]. Our study revealed that 4MU treatment significantly modified the expression of two NR1H4-dependent genes, namely Cyp7a1 and Hhex. This finding is consistent with the PubChem BioAssay data, which clearly states that 4MU is a non-specific ligand for nuclear receptors, including NR1H4. We also observed an indubitable downregulation of immune-associated genes involved in interferon signalling.

PubChem BioAssay database revealed high-affinity specific inhibition of HSD17b3, HSD17b4, and HSD17b10 belonging to the HSD17B family of NAD(P)H/NAD(P)+-dependent short-chain oxidoreductases. HSD17B1, HSD17B2, HSD17B3, HSD17B5, and HSD17B6 catalyse the interconversion between less potent 17-ketosteroids and more potent 17-hydroxysteroids to maintain the balance between 17-keto/17β-hydroxy forms of estrogens and androgens [116]. Other members of this family are involved in fatty acid metabolism, cholesterol biosynthesis, and bile acid production [117]. One member of this family, HSD17B13, is of paramount interest as a potential target for drug development. HSD17B13 was first cloned from the human liver cDNA library [118]. It was later shown that the HSD17B13 genetic polymorphism rs72613567, causing splicing alteration and loss of HSD17B13 activity, is associated with a substantial decrease in serum alanine aminotransferase and aspartate aminotransferase levels in a European population study. This polymorphism was also associated with a lower risk of NAFLD and non-alcoholic cirrhosis [119]. These findings were later confirmed in an independent study by Pirola, C.J. et al. [120]. Although data on 4MU inhibition of HSD17B13 are missing from the PubChem BioAssay database, the high homology of HSD17 family members and inhibition of b3, b4 and b10 by low micromolar concentration of 4MU allow us to infer the possible inhibition of HSD17b13 activity by 4MU, which may contribute to its protective effect in liver fibrosis [6].

Inhibition of KAT2A by 4MU in low micromolar concentrations (Table 2) underpins the recent discovery of its involvement in biliary fibrosis in mice. KAT2A, also known as GCN5, is a histone acetyltransferase (HAT) that functions primarily as a transcription activator. It also represses NFkB activity by promoting the ubiquitination of the NFkB subunit RelA (p65) in a HAT-independent manner [121]. Both pharmacologic inhibition of KAT2A lysine acetyltransferase activity and cholangiocyte-specific deletion of KAT2A were protective in mouse models of biliary fibrosis [122].

TGF-β ligands that activate the SMAD-2/3 intracellular pathway are considered a major driver of human fibrotic pathologies [123,124,125]. The involvement of the TGF-β/SMAD3 pathway is well documented in hepatic stellate cell activation and collagen production [124]. SMAD3 signalling was mechanistically linked to the liver fibrosis development in mice. Specifically, the anti-fibrotic action of Umbelliferon, a close analogue of 4MU, was associated with inhibition of TGF-β SMAD3 signalling [126]. SMAD3 inhibition by 4MU was reported in four independent bioassays (Table 2). Together, these data justify the validity of our bioinformatic approach, which independently identified SMAD3 as a potential 4MU target.

By its nature the big data analysis presented here only suggested potential targets of 4MU which have to be individually validated in our ongoing research using in vitro and in vivo techniques, including gene knockout and knockdown.

## 4. Materials and Methods

**Data collection.** Publicly available data were collected from the PubChem BioAssay (U.S. National Library of Medicine, Bethesda, MD, USA) [28] database for the next CIDs: 5280567 (Hymecromone, 4MU), 84843 (4-methylumbelliferone sulfate, MUS), 91553 (4-methylumbelliferone glucuronide, MUG). Data were downloaded manually, and all subsequent data manipulations were performed with a custom-made R script in RStudio version 1.4.1106, R version 3.6.3 (Posid, Boston, MA, USA). Briefly, the collected dataset containing 1762 rows was filtered according to substance activity. Individual observations without target names (calibration controls) were excluded from further analysis. In the final step, we identified common 4MU targets and its derivatives based on protein name. Expression profiles of 4MU target proteins in different cell types were manually downloaded from the Human protein atlas database [127] (https://www.proteinatlas.org/; accessed on 3 June 2022) from Single Cell and HPA datasets.

**Mice experiments.** All animal procedures were conducted following the Russian Academy of Science Guidelines for Animal Experimentation and were approved by the Institute of Developmental Biology RAS Ethics committee. 8-week-old female Balb/c mice (18–20 g.) were obtained from “Scientific Centre for Biomedical Technologies” of the Federal Medical and Biological Agency, Moscow, Russia. Mice were fed a diet of natural ingredients and housed in a 12-h light/dark cycle with an ad libitum access to food and water at a room temperature of 22 °C and relative humidity of 55–65%. Mice were allowed to acclimatise to the conditions for 2–3 days prior to the experiment. The 4MU was purchased from Sigma-Aldrich (Cat # M1381, St. Louis, MO, USA), mixed with 0.5% methylcellulose, and administered per os through gavage at a concentration of 600 mg/kg daily for 2 or 4 weeks in total. To the control mice, 5% methylcellulose slurry was administered via the same route. At the end of the experiment, animals were terminally anaesthetised with 5% isoflurane and humanely sacrificed by rapid exsanguination followed by cervical dislocation. Liver samples were collected for RNA isolation and histological examination.

**Liver histology and staining.** Freshly isolated liver samples were cut into 3 × 3 mm pieces and fixed in 10% buffered formalin for 24 h. After an extensive wash in diH2O, samples were dehydrated in isopropanol solutions with rising concentrations from 70% to 100%, followed by two immersions in xylene and then embedded in Histomix (Biovitrum, Russia) at 56 °C. Embedded tissue samples were sectioned by microtome at 5 µm slices and mounted onto SuperFrost glass slides. We used commercially available kits for Pass-Shiff and Gomori-Grocott staining (Biovitrum, Russia). The images of histological specimens were taken with microscope Keyence BZ9000 BioRevo (Yokogawa Electric Corporation, Tokyo, Japan).

**RNA isolation and library preparation.** TotRNA was isolated from liver tissue samples (60–100 mg) using TRI Reagent (Sigma-Aldrich, Merck KGaA, Darmstadt, Germany) according to the manufacture protocol. 3–5 μg of totRNA was used for isolated mRNA by NEBNext Poly(A) mRNA Magnetic Isolation Module (New England BioLabs, MA, USA) according to the manufacture protocol. RNA concentration was measured by Nanodrop (Thermo Fisher Scientific, MA, USA), and RNA samples were analysed by capillary electrophoresis using an Agilent capillary electrophoresis system (Agilent, CA, USA). cDNA libraries were constructed using NEBNext Ultra II Directional RNA Library Prep Kit for Illumina (New England BioLabs, MA, USA) following the manufacturer’s protocol. cDNA libraries were sequenced using the NextSeq500 (Illumina, San Diego, CA USA) instrument. Hence, 33–41 million raw reads were obtained for each sample with a 75-bp read length.

**RNASeq data analysis.** Raw reads were preprocessed as follows: trimmed with Trimmomatic (v0.39) [124] to remove the adapters. The processed reads were aligned to the Mus musculus genome (assembly GRCm39.105) using the HISAT2 algorithm [128]. Follow analysis was performed in R version 3.4.2. Gene read counts were calculated using Rsubread package, function feature Counts (with parameters countMultiMappingReads = F, isPairedEnd = TRUE) [129]. Genes with less than 10 reads on average for each sample were filtered out. To check the data for self-consistency, the following analysis was carried out: correlation analysis and MDS using the Spearman correlation and PCA using normalised gene counts as CPM (count per million). DEGs were identified using edgeR package version 3.20.9 [130] as follows. First, the read counts were normalised using the calcNormFactors function (RLE algorithm). Genes differentially expressed between each group of samples and appropriate control were identified using the estimateDisp, glmFit, and glmLRT functions with a 0.05 FDR significance threshold [131]. Hierarchical clustering was carried out to identify the modules of genes using hclust and cutree functions. Gene ontology analysis of DEGs modules was performed using the ClusterProfiler package with all significant genes as background [132]. Pathway enrichment analyses were performed across Bioplanet 2019, GO Molecular function [133,134], MSigDB_Hellmark_2020, and KEGG_Pathways_2019 CHEA 2016 databases to annotate differences in molecular functions, biological processes, and signalling pathways between experimental groups with Enrichr (http://amp.pharm.mssm.edu/Enrichr; New York, NY, USA).

## 5. Conclusions

Our study presents multiple biological targets of 4MU, including enzymes, nuclear receptors, and transcription factors. Detailed investigation of unravelled 4MU targets presented here will be pivotal in the discovery of new drugs for the efficient treatment of pathological processes associated with energy metabolism, inflammation, carcinogenesis, and tissue repair. Our transcriptomic data support the public data indicating nuclear receptors as the main targets of 4MU action. Future work will allow us to validate these targets using in vivo gene knockdown techniques in experimental models of fibrosis as well as immunological and neurodegenerative disorders.

## Figures and Tables

**Table 1:** Function of the acquired 4MU target proteins.

Protein	Full Name	Function	Ref.
Enzymes			
ACHE	Acetylcholinesterase	Primary cholinesterase of the human body which breaks down the neurotransmitter acetylcholine	[29]
AKR1B1	Aldo-keto reductase family 1 member B1	Catalyses the NADPH-dependent reduction of a wide variety of carbonyl-containing compounds to their corresponding alcohols. Displays enzymatic activity towards endogenous metabolites such as aromatic and aliphatic aldehydes, ketones, monosaccharides, bile acids and xenobiotics substrates. The overexpression of AKR1B1 is associated with inflammation and carcinogenesis	[30]
ALDH1A1	Aldehyde dehydrogenase 1A1	Is the second enzyme of the major oxidative pathway of alcohol metabolism, oxidise retinal to retinoic acid. The enzyme involved in adipogenesis, glucose tolerance and abdominal fat formation	[31]
CA9, CA12	Carboxylic anhydrase 9 and 12	Zinc metalloenzyme catalyse CO_2_ hydration to bicarbonate and H^+^ and regulate pH level, acid-base homeostasis and fluid balance. Expression induced by hypoxia and implicated in cancer development and adaptation to acidosis.	[32,33,34]
CASP1	Caspase 1	Is a part of the inflammasome complex, which initiates a proinflammatory response through the cleavage of IL1B and IL18	[35,36,37]
CASP3	Caspase 3	Involved in the activation of apoptosis, sterol regulatory element-binding proteins (SCREBPs) and CASP7	[38]
CASP7	Caspase 7	Involved in activation of apoptosis	[38]
CYP1A2	Cytochrome P450 1A2	Involved in the metabolism of aromatic compounds, xenobiotics, drugs and steroids	[39,40]
GAA	Acid alpha-glucosidase	The key enzymes of lysosomal glycogen breakdown.	[41]
GALC GBA GLA	Galactocerebroside beta Glucosylceramidase beta Alpha-galactosidase A	Involved in the glycosamide degradation in lysosomes and membrane turnover	[42]
HPGD	15-Hydroxyprostaglandin dehydrogenase	Catalyses the oxidation of a broad array of hydroxylated polyunsaturated fatty acids (mainly eicosanoids and docosanoids, including prostaglandins, lipoxins and resolvins), yielding their corresponding keto (oxo) metabolites	[43]
HSD17B10	17-β-Hydroxysteroid dehydrogenase 10	Involved in steroid metabolism, Isoleucine and branched-chained fatty acids mitochondrial oxidation.	[44,45]
HSD17B3	17β-Hydroxysteroid dehydrogenase 3	Convert androstenedione to testosterone in testis	[46,47]
HSD17B4	17β-Hydroxysteroid dehydrogenase 4	Involved in peroxisomal beta-oxidation of fatty acids and fatty acid derivatives	[48]
KAT2A	Histone acetyltransferase KAT2A	Catalyses succinylation of histone H3 with a maximum frequency around the transcription start sites of genes	[49]
KLK7	Kallikrein-related peptidase 7	Involved in the continuous shedding of cells from the skin surface and insulin proteolytic cleavage, regulation of body weight, energy metabolism, insulin sensitivity and obesity-associated adipose tissue dysfunction	[50,51]
MAPK1	Mitogen-activated protein kinase 1	Also known as ERK2 and regulates diverse cellular programs by relaying extracellular signals to intracellular responses	[52]
TDP1	Tyrosyl-DNA phosphodiesterase 1	Hydrolyses the phosphodiester bond between a DNA 3′ end and a tyrosyl moiety and is involved in double break DNA repair	[53]
*TXNRD1*	Thioredoxin reductase 1	Involved in actin polymerisation and membrane protrusion formation, estrogen receptor signalling, enhances apoptosis in cancer cells after INF gamma and retinoic acid treatment	[54]
Transcription factors			
AR ESR1 ESR2 ESRRA PGR	Androgen receptor Estrogen receptor alpha Estrogen receptor beta Estrogen-related receptor alpha Progesterone receptor	Nuclear receptors regulate sex-dependent steroid hormone signalling and modulate the immune response, adipose tissue metabolism, insulin sensitivity, and glucose homeostasis.	[55,56,57]
ATF6	Activating transcription factor 6	The key TF controls the protein quality in the endoplasmic reticulum. It interacts with the PPARA-RXR complex and regulates liver fatty acid oxidation and glucose metabolism.	[58,59,60]
GLI3	Zinc finger protein GLI3	Mediates Sonic hedgehog signalling in many tissues. It represses lipid synthesis in the liver at a transcriptional level, and disruption of this pathway leads to steatosis. It is involved in immune cell development and maturation.	[61,62]
HIF1A	Hypoxia-inducible factor 1-alpha	The primary sensor of oxygen supply. Activation of the HIF-pathway shifts glucose metabolism from oxidative phosphorylation to glycolysis.	[63]
NFE2L2	Nuclear factor erythroid 2-related factor 2	Involved in immediate reaction to oxidative stress, hypoxia, toxins and infections	[64]
NFkB	Nuclear factor NF-kappa-B p105 subunit	The key TF orchestrates inflammatory responses to different stimuli.	[65]
NR1H4	Farnesoid X receptor	The primary receptor for bile acids, which is involved in the regulation of bile acid synthesis and degradation, glucose and fatty acid metabolism	[66,67,68]
NR1I3	Constitutive androstane receptor	The primary xenobiotic sensor. Natural ligands include androstanes and their derivatives. It regulates the expression of genes involved in drug and xenobiotic transformation and excretion. Its activation inhibits gluconeogenesis in hepatocytes and stimulates their proliferation.	[69]
NR3C1	Glucocorticoid receptor	Mediates cortisol and others glucocorticoids effects and controls genes involved in the development, metabolism and immune response.	[70]
PPARD PPARG	Peroxisome proliferator-activated receptor delta Peroxisome proliferator-activated receptor gamma	The nuclear receptors for free fatty acids, eicosanoids and vitamin B3. They have a central role in the regulation of glucose and lipid homeostasis	[71]
RXRA	Retinoid X receptor alpha	Serves as the main heterodimerisation partner for type II nuclear receptors (LXR, PPARs, VDR, NR1H4 and NR1I2). RXRA is involved in regulating fatty acid and cholesterol metabolism, innate immunity and cellular senescence.	[72,73]
SMAD2 SMAD3	Mothers against decapentaplegic homolog 2 Mothers against decapentaplegic homolog 3	The Smad-signaling pathway, which operates downstream of the transforming growth factor-β (TGF-β) superfamily of ligands, regulates a diverse set of biological processes, including proliferation, differentiation and apoptosis, in many different organ systems	[74,75,76]
TP53	Tumour protein P53	Plays a fundamental role in regulating the cell cycle, apoptosis, and genomic stability.	[77]
VDR	Vitamin D receptor	The primary regulator of Ca metabolism and immune response.	[78,79]
Others			
ABCG2	ABC transporter G2	The main transporter of physiological compounds, dietary toxins, xenobiotics and drugs outside the cells.	[80]
H2AX	H2A histone family member X	Histone which is involved in double break DNA repair, chromatin remodelling and nuclear envelope stabilisation	[81]
HSPB1	Heat shock protein 27	The main molecular chaperon which involved in protein refolding under stress conditions.	[82]
HTT	Huntingtin	A protein which underlines Huntington’s disease development and usually is involved in axonal transport	[83]
KPNB1	Importin subunit beta-1	The nuclear importin mediates the transport of various proteins into the cell nucleus.	[84]
LMNA	Lamin A/C	Nuclear Lamin A is a scaffold protein of the inner nuclear envelope, which regulates nuclear membrane stability and inner nuclear architecture	[85,86]
PMP22	Peripheral myelin protein 22	Comprise 2–5% of peripheral myelin proteins and critical for nerve membrane ultrastructure	[87,88]
SNUPN	Snurportin1	The protein mediates (m3G)-cap-dependent nuclear import of spliceosomal RNA-protein complexes	[89]

**Table 2:** 4MU active concentration and effect on target protein function.

Effect	Protein Type	Target Name	Acname	Acvalue, μM	AID	qHTS Assay
Activation	Enzymes	CASP3	NULL	24.35	463141	Absorbance-based
		GAA	Potency	25.12	2112	Fluorescence-based
		GALC	Potency	28.18	1159614	Fluorescence-based
		GBA	Potency	14.13	2101	Fluorescence-based
		KLK7	NULL	7	652039, 686949	Fluorescence-based
	TFs	ATF6	Potency	40.72	1159516, 1159519	Fluorescence-based
		HIF1A	Potency	46.22–48.97	1224846, 1224894	Fluorescence-based
		NFE2L2	Potency	33.49–61.13	743202, 743219	FRET-based
		NFkB	IC50	24.54	1159509, 1159518	Fluorescence-based
		RXRA	Potency	22.32	1159527	Fluorescence-based
		TP53	Potency	61.13	651631, 720552	FRET-based
	Others	H2AX	Potency	39.08–89.76	1224845	TR-FRET-based
Inhibition	Enzymes	AKR1B1	IC50	24.5	516862	Absorbance based
		ALDH1A1	Potency	15.85	1030	Fluorescence-based
		ACHE	Potency	6.17–89.36	1347398	Fluorescence-based
		CA12	Ki	8.1	641826	Colorimetric CO_2_ Hydration
		CA9	Ki	0.56	641825	Colorimetric CO_2_ Hydration
		CASP1	Potency	12.59	900	Fluorescence-based
		CASP7	Potency	12.59	889	Fluorescence-based
		CYP1A2	NULL	3.98	1851, 410	Luciferase-based
		GLA	Potency	19.95–28.18	1467, 2107	Fluorescence-based
		HPGD	Potency	35.48	894	Fluorescence-based
		HSD17B3	IC50	1	452459	Cell-based assays
		HSD17B4	Potency	10	893, 886	Fluorescence-based
		HSD17B10	Potency	2.51		
		KAT2A	Potency	11.22	504327	Fluorescence-based
		MAPK1	Potency	39.81	995	AlphaScreen-Based
		TXNRD1	Potency	50.12	588453	Fluorescence-based
		TDP1	Potency	20.6	686978	Luciferase-based
	TFs	GLI3	Potency	15.36–29.48	1259369, 1259392	Luciferase-based
		NR1I3	Potency	43.28	1224893	Luciferase-based
	Others	KPNB1	Potency	70.8	540263	FRET-based
		SNUPN	Potency	70.8		
Both	TFs	AR	Potency	20.04	743054, 743036, 743035, 1259243, 743040	FRET-based, Fluorescence-based, Luciferase -based
		ESR1	Potency	30.9–54.48	743069, 743079	FRET-based
		ESR2	Potency	23.54	1259377,1259378, 1259394	FRET-based
		ESRRA	Potency	13.69	1224848,1259403,1259404	Luciferase-based
		NR1H4	Potency	38.15	743217, 743220, 743239	FRET-based
		NR3C1	Potency	43.64–48.97	720691, 720725, 720719	FRET-based
		PGR	Potency	48.56	1346784, 1346795, 1347031	FRET-based
		PPARD	Potency	35.14	743226, 743212, 743227,743215	FRET-based
		PPARG	Potency	39.57	743191, 743094, 743140, 743199	FRET-based
		SMAD2	Potency	48.97	1346859, 1346924, 1347032, 1347035	FRET-based
		SMAD3	Potency	48.97		
		VDR	Potency	28.71	743223, 743242, 743222, 743241	FRET-based

Acvalue was calculated as the mean, if the number of AID was at least 3. Potency is a concentration at which a compound exhibits half-maximal efficacy; a name = NULL means what compound was active in the mentioned concentration. According to PubChemBioAssays to each tested compound, the activity normalized score is assigned (PUBCHEM_ACTIVITY_SCORE) between 0 and 100 where the most active result has scores closer to 100 and inactive closer to 0. Active compounds have PUBCHEM_ACTIVITY_SCORE between 40 and 100. Inconclusive compounds have PUBCHEM_ACTIVITY_SCORE between 1 and 39 (https://pubchem.ncbi.nlm.nih.gov/upload/html/tags_assay.html; accessed on 30 May 2022). Data normalization and activity estimation are the part of the standard data table in PubChemBioAssays repositorium. Acname—the data base name of measured activity type: IC50, EC50, Kd, Ki. Acvalue μM—estimated compound concentration of Acname. Acvalue—as mean of individual Acval if the number of identified assays (AID) was more than 2. AID—the accession ID number in the database. qHTS assay—type of High throughput screens assay.

**Table 3:** Top 52 genes differentially expressed in the liver of mice fed with 4MU.

Gene Name	Gene ID	Description	Function
Down regulated			
Ly6d	17068	lymphocyte antigen 6 complex locus D	Surface markers of immune cells
Ly6c1	17067	lymphocyte antigen 6 complex locus C1	
Themis2	230787	thymocyte selection associated family member 2	
Ms4a4b	60361	membrane spanning 4 domains subfamily A member 4B	
Tgtp1	21822	T cell specific GTPase 1	Involved in interferon signalling and response
Mx1	17857	MX dynamin like GTPase 1	
Epsti1	108670	epithelial stromal interaction 1 (breast)	
Ifit1	15957	interferon induced protein with tetratricopeptide repeats 1	
Ifit3	15959	interferon induced protein with tetratricopeptide repeats 3	
Ifit3b	667370	interferon induced protein with tetratricopeptide repeats 3B	
Gbp3	55932	guanylate binding protein 3	
Isg15	100038882	ISG15 ubiquitin like modifier	
Il18bp	16068	interleukin 18 binding protein	
Usp18	24110	ubiquitin specific peptidase 18	
Irf7	54123	interferon regulatory factor 7	
Oasl2	23962	2′ 5′ oligoadenylate synthetase like 2	
Ifi27l2a	76933	interferon alpha inducible protein 27 like 2A	
Oas1a	246730	2′ 5′ oligoadenylate synthetase 1A	
Naip1	17940	NLR family apoptosis inhibitory protein 1	
Cdc20	107995	cell division cycle 20	involved in cell division and DNA stability
Mki67	17345	antigen identified by monoclonal antibody Ki 67	
Ccn5	22403	cellular communication network factor 5	
Racgap1	26934	Rac GTPase activating protein 1	
Top2a	21973	topoisomerase (DNA) II alpha	
Blm	12144	Bloom syndrome RecQ like helicase	
Nfe2	18022	nuclear factor erythroid derived 2	TF—regulated maturation, differentiation and epithelial-mesenchymal transition
Snai2	20583	snail family zinc finger 2	
Hba-a2	110257	hemoglobin alpha adult chain 2	Involved in oxygen transport
Hbb-b1	15129	hemoglobin beta adult major chain	
Hbb-bt	101488143	hemoglobin beta adult t chain	
Rbp1	19659	retinol binding protein 1 cellular	involved in the transport of retinol
Ighd	380797	immunoglobulin heavy constant delta	antigen and immunoglobulin receptor binding activity
Slc41a3	71699	solute carrier family 41 member 3	mitochondrial Mg^2+^ transporter
Cyp7a1	13122	cytochrome P450 family 7 subfamily a polypeptide 1	enzymes of bile acid production
Up regulated			
Bcl3	12051	B cell leukemia/lymphoma 3	involved in the modulation of transcriptional activity, histone acetylation
Spen	56381	spen family transcription repressor	
Dot1l	208266	DOT1 like histone H3 methyltransferase (S. cerevisiae)	
Kmt2d	381022	lysine (K) specific methyltransferase 2D	
Dusp6	67603	dual specificity phosphatase 6	
Ppp1r10	52040	protein phosphatase 1 regulatory subunit 10	
Lars2	102436	leucyl tRNA synthetase mitochondrial	mitochondrial biogenesis
Pprc1	226169	peroxisome proliferative activated receptor gamma coactivator related 1	
Egfl7	353156	EGF like domain 7	extracellular matrix remodelling
Ntn3	18209	netrin 3	
Papln	170721	papilin proteoglycan like sulfated glycoprotein	
Myoz2	59006	myozenin 2	cells differentiation
Tmod4	50874	tropomodulin 4	
Sez6l2	233878	seizure related 6 homolog like 2	
Hhex	15242	hematopoietically expressed homeobox	TF—regulated of differentiation, inflammatory responses and metabolism
Jun	16476	jun proto oncogene	
Nfkbiz	80859	nuclear factor of kappa light polypeptide gene enhancer in B cells inhibitor zeta	
Zbtb16	235320	zinc finger and BTB domain containing 16	

## Data Availability

The data analysed in the study are publicly available in PubChem BioAssay database with the following CIDs: 5280567 (Hymecromone, 4MU), 84843 (4-methylumbelliferone sulfate, MUS), 91553 (4-methylumbelliferone glucuronide, MUG). The Rna-Seq dataset is available from corresponding author upon request.
